# Delusional Infestation

**DOI:** 10.1155/2016/9091838

**Published:** 2016-03-29

**Authors:** Kevin B. Laupland, Louis Valiquette

**Affiliations:** ^1^Department of Medicine, Royal Inland Hospital, Kamloops, BC, Canada V2C 2T1; ^2^Departments of Medicine, Critical Care Medicine, Pathology and Laboratory Medicine, and Community Health Sciences, University of Calgary, Calgary, AB, Canada; ^3^Department of Microbiology-Infectious Diseases, Université de Sherbrooke, Sherbrooke, QC, Canada J1H 5N4

## Abstract

Although the practice of infectious diseases involves a broad range of surgical and medical disciplines, interactions with psychiatry are infrequent. Delusional infestation is a condition where an individual has a firmly fixed false belief that they have an infection. Delusional infestation challenges the infectious diseases specialist who must diligently rule out the presence of a true infection. However, perhaps, more importantly, we may need to initiate therapy with neuroleptic medications for which we may have little specific knowledge and experience. In this note we review the diagnosis and management of patients with delusional infestation.

## 1. Definition

Delusional infestation is a condition where an individual has a firmly fixed false belief that they are infested with an infectious agent. Delusional infestation is preferred to the narrower term of delusional parasitosis as patients may report being affected by a range of nondescript or specific vermin, insects, parasites, fungi, bacteria, or viruses [1–6]. While the delusional symptomatology may include any body system, most frequently patients will report formication or sensations of movement or other phenomena associated with the skin.

While it is not uncommon for individuals from time to time to believe that they may have an infection, this must be differentiated from the case where this belief is intrusive and persists despite a lack of compelling supportive evidence. With the exception of thoughts and behaviors directly related to the delusion, patients may be otherwise composed and highly functional.

A diagnosis of delusional infestation requires persistence of the delusion for at least one month and may be further classified into either primary or secondary. In primary delusional infestation a patient will have no underlying or prior psychiatric disorder and the delusion is not related to an organic cause or substance abuse. Secondary delusional infestation is related to either an underlying psychiatric or medical disorder or substance abuse. Underlying psychiatric disorders may include schizophrenia or depression [7]. A broad range of medications as well as dermatologic, endocrine, neurological, hematologic, renal, cardiovascular, and infectious diseases have been associated with secondary delusional infestation [8, 9]. Alcohol, cocaine, methamphetamines, tetrahydrocannabinol, and other illicit substances have been associated with secondary delusional infestation [10].

## 2. Epidemiology

Delusional infestation is considered to be a relatively uncommon condition. Case reports and series have been reported from most regions from around the globe. Freudenmann and Lepping reviewed observational studies of larger than 10 patients from 1896 to 2008 and reported that, among more than 1400 included patients, the ratio of females to males was 1.3–5.7 : 1, the age range was 17–92 years, and the average duration of illness was approximately 3 years [5]. Since the publication of their review a number of other case series have been published [11–14].

Few studies have estimated or established the occurrence of delusional infestation at the population level. Survey studies conducted in Germany, California, and the United Kingdom have estimated prevalence rates ranging from 0.18 to 4.2 per 100,000 of the population [15–18]. Bailey et al. conducted a population-based study in Olmsted County, Minnesota, during 1976 and 2010 [18]. They found an overall annual incidence rate of 1.9 per 100,000 of the population, with higher rates observed with older age and in most recent years of the study. These data indicate that while delusional infestation is not common, it is certainly not rare.

## 3. Clinical Considerations

Although the skin is most commonly involved, symptomatology may range in any body system. While a detailed review of all of these is beyond the scope of this review, there are some clinical considerations that are particularly noteworthy.

Patients with delusional infestation will frequently present with a range of artifacts in an effort to provide proof of infestation known as the “specimen” or “matchbox” sign [19, 20]. Typically patients will present small containers (of which there may be many) or bags that may include a range of patient related specimens (i.e., hair, bits of skin or scabs), fibers and threads, innocuous environmental materials such as sand or dirt, or even parts of insects and other creatures. Digital photographs and media represent the modern version of this sign first recognized more than 100 years ago [21]. Specimens are often presented without apprehension or disgust and may be accompanied with elaborate descriptions related to symptomatology and “pathogenesis” related to the purported route of infestation.

Another important clinical consideration is the potential for associated harmful behaviors resulting from attempts to eradicate the infestation. Patients may attempt to physically remove the infestation from their bodies. Resulting damage to skin, hair, eyes, genitals, and other body parts is well described [4, 22, 23]. Secondary infections may complicate self-mutilation behaviors and may be the inciting event that leads to referral to an infectious disease specialist. Ingestion of medication or other agents by patients aimed at treating the infestation may be toxic. Attempts to decontaminate the environment through application of chemicals or fire poses risk to both themselves and others. Accordingly, awareness for the well-being of children and other dependents should be a consideration.

Shared delusional infestation is estimated to occur in approximately 5–15% of cases [24]. In* folie à deux*, two individuals may concomitantly share a diagnosis of delusional infestation [25]. Cases of three or more concomitant patients with delusional infestation have been reported. Interestingly, secondary cases may respond well to either separation from or treatment of the index case [24]. In delusional infestation* by proxy*, individuals believe that other individuals or pets are infested [26].

There are a number of other miscellaneous clinical issues that merit brief notation. Diagnosis of delusional infestation may follow the successful treatment of a patient with a documented infection and may be confused with reinfection or relapse. Delusional infestation has been recognized as a potential occupational hazard of being an entomologist or healthcare worker [27]. Iatrogenic cases of delusional infestation have been reported [28, 29]. Morgellons syndrome is an entity whereby patients report fibers or other solid materials coming out of the skin in association with skin lesions and sensory phenomena. While it has been debated as to whether this represents a discrete entity, it is currently viewed within the spectrum of delusional infestation [16, 30].

## 4. Approach to Management

The first and foremost responsibility of the infectious diseases physician is to rule out the presence of an infection. Clinical assessment and diagnostic investigation should be directed initially to the presenting complaint (i.e., evaluation of the skin in patients who present with formication). However, other infections such as syphilis, tuberculosis, Lyme disease, and HIV should be considered potential secondary causes. A review of systems, medication and substance use review, general medical assessment, and psychiatric history review should be undertaken to explore the possibility of a noninfectious underlying disorder.

Management of infectious and noninfectious secondary causes of delusional infestation represents the initial step of therapy. However, in the absence of an identifiable secondary etiology, neuroleptic treatment is indicated. While debate exists because optimal agents and clinical trials are lacking, commonly recommended therapies include risperidone, olanzapine, and aripiprazole [31–33]. Therapeutic effect may be observed within 1-2 weeks with maximal effect occurring within 3–10 weeks [31]. Remission is expected to occur in 75% of patients. Twenty-five percent of successfully treated patients may relapse following neuroleptic withdrawal [34].

A number of recommendations have been published with respect to the appropriate dialogue and means to engage patients in therapy [35, 36]. Importantly, while it may be tempting to offer a therapeutic trial with an antiparasitic or antimicrobial in an effort to try to convince the patient that they will be cured, this typically has a paradoxical effect and further reinforces false beliefs of infestation [5]. Patients referred to infectious diseases specialists will often have already had a number of these unsuccessful therapeutic trials. Patients will not usually be initially receptive to psychiatrist referral and as such infectious diseases physicians may need to be the initial prescribers of neuroleptics. One approach to increase the initial acceptance of neuroleptics is to offer these medications not necessarily as a means to cure the patient's disease* per se* but rather to help them cope with the symptoms.

## Additional Points

The following is a summary of the paper. Delusional infestation is a relatively uncommon but not rare condition that will often be referred to infectious diseases specialists for assessment and management. Recognition of the possibility of this condition and a careful search for secondary causes are critical. While we may seek assistance from our psychiatrist colleagues, we must accept the fact that initial referral of patients with delusional infestation is rarely accepted by patients. Infectious diseases physicians will need to step out of their comfort zone and accept the fact that we will need to be the initial prescribing physicians for neuroleptic therapy. Further efforts are needed to define the scope of the problem, create awareness among medical professionals, and optimize pharmacologic and other therapeutic interventions in the Canadian context.
